# ZIKV Strains Differentially Affect Survival of Human Fetal Astrocytes versus Neurons and Traffic of ZIKV-Laden Endocytotic Compartments

**DOI:** 10.1038/s41598-019-44559-8

**Published:** 2019-05-30

**Authors:** Jernej Jorgačevski, Miša Korva, Maja Potokar, Marjeta Lisjak, Tatjana Avšič-Županc, Robert Zorec

**Affiliations:** 1 Laboratory of Neuroendocrinology – Molecular Cell Physiology, Institute of Pathophysiology, Faculty of Medicine, University of Ljubljana, Zaloška 4, 1000 Ljubljana, Slovenia; 2 Institute of Microbiology and Immunology, Faculty of Medicine, University of Ljubljana, Zaloška 4, 1000 Ljubljana, Slovenia; 3 Celica BIOMEDICAL, Tehnološki park 24, 1000 Ljubljana, Slovenia

**Keywords:** Endocytosis, Pathogens, Viral host response, Cellular neuroscience, Astrocyte

## Abstract

Malformations of the fetal CNS, known as microcephaly, have been linked to Zika virus (ZIKV) infection. Here, the responses of mammalian and mosquito cell lines, in addition to primary human fetal astrocytes and neurons were studied following infection by ZIKV strains Brazil 2016 (ZIKV-BR), French Polynesia 2013 (ZIKV-FP), and Uganda #976 1947 (ZIKV-UG). Viral production, cell viability, infectivity rate, and mobility of endocytotic ZIKV-laden vesicles were compared. All cell types (SK-N-SH, Vero E6, C6/36, human fetal astrocytes and human fetal neurons) released productive virus. Among primary cells, astrocytes were more susceptible to ZIKV infection than neurons, released more progeny virus and tolerated higher virus loads than neurons. In general, the infection rate of ZIKV-UG strain was the highest. All ZIKV strains elicited differences in trafficking of ZIKV-laden endocytotic vesicles in the majority of cell types, including astrocytes and neurons, except in mosquito cells, where ZIKV infection failed to induce cell death. These results represent a thorough screening of cell viability, infection and production of three ZIKV strains in five different cell types and demonstrate that ZIKV affects vesicle mobility in all but mosquito cells.

## Introduction

Last Zika virus (ZIKV) epidemics in French Polynesia, South and Central America, and the Caribbean have been accompanied by an increase in the incidence of different neurologic disorders in adults, such as the Guillain-Barré syndrome, myelitis, encephalitis, and neuralgia^1–3^. However, it is particularly worrying that ZIKV may be transmitted from an infected mother to her fetus, causing deleterious consequences, such as fetal growth restriction, death, and several abnormalities of the CNS^4–9^. Macroscopic pathologic changes in ZIKV-infected brain are first observed at the mid-gestation period and include microcephaly, cerebral atrophy, small cerebellum and brain stem, agyria, hydrocephalus, and calcifications in the neocortex and the subcortical white matter^4,5,7^. These features reflect the fact that the physiology and/or morphology of ZIKV-infected neural cells in the fetus must have been altered at some point after infection. For example, activated microglial cells and macrophages, diffused reactive astrocytes, and granular ZIKV assemblies in neuronal structures have been detected in ZIKV-infected fetal brain^4,5^. However, how the function of infected cells is altered remains an open question. In general, ZIKV infection of neuronal and skin cells can arrest cell-cycle progression, affect differentiation and proliferation, and appears to alter the immune response and cell death, which together likely contribute to arrest fetal brain growth^8,10–14^.

Growth of the fetal cerebral cortex continues throughout fetal development, but at the mid-gestation period particularly, extensive neurogenesis and gliogenesis occur^15–18^. At around gestation week (gw) 20, ZIKV-infected fetuses show severely hampered brain development, evident as a reduction in brain size^6^. By this time, the astrocyte population is already abundant and their infection may contribute to further expansion of microcephaly, which may have originated in ZIKV infection of human neural progenitor cells (NPCs), neural stem cells (NSC), or radial glia cells (RGC) in which ZIKV infection has already been confirmed^19,20^. Astrocytes have many critical roles in the functioning of neurons; e.g., they provide metabolic support for neuronal networks, integrate synaptic transmission, modulate the integration of neurons into networks, and regulate synaptogenesis, brain microcirculation, the integrity of the blood-brain barrier^21^ and immune response^22–26^, which may consequently all be affected by ZIKV infection. The dynamics of vesicle trafficking, one of several processes in astrocytes that affect neuronal function, is altered in numerous pathologic conditions^24,27–32^. While it is currently unknown whether ZIKV infection alters vesicle traffic, the infection-induced changes in different trafficking factors^33^ and reorganization of the cytoskeleton^34^, indicate that this might be the case.

In this study, we tested the hypothesis that astrocytes are the key target for ZIKV infection, as reported for another member of the *Flaviviridae* family, tick-borne encephalitis virus (TBEV), which affects astrocytes in the rodent brain to become a reservoir for TBEV^35–37^. We report how human astrocytes respond to ZIKV infection in terms of the extent of viral infection, release of productive virus, host cell survival, and intracellular traffic of ZIKV-laden endocytotic vesicles. We compared their responses to neurons and to selected mammalian and mosquito cell lines.

## Results

During the ZIKV epidemics in Micronesia (2007), French Polynesia (FP, 2013–2014), other South Pacific islands (2014–2015), and in Latin America and the Caribbean (BR, 2015–2016), strains originating from the Asian ZIKV lineage were found circulating in the human population^1,2^. Here, we used two Asian-lineage ZIKV strains ZIKV-FP (2013) and ZIKV-BR (2016), which are responsible for the latest international outbreaks causing neuropathology^13^, and the African lineage ZIKV-UG #976 (1947), to test their infectivity rate, release, effects on cell viability, cytopathologic effects (CPE), and the mobility of ZIKV-laden endocytotic vesicles in mammalian (SK-N-SH and Vero E6) and mosquito (C6/36) cell lines, as well as in human fetal astrocytes and neurons.

### The infectious virus production in mammalian cell lines is similar to mosquito cell line that resist ZIKV-Triggered cell death

**Figure 1:**
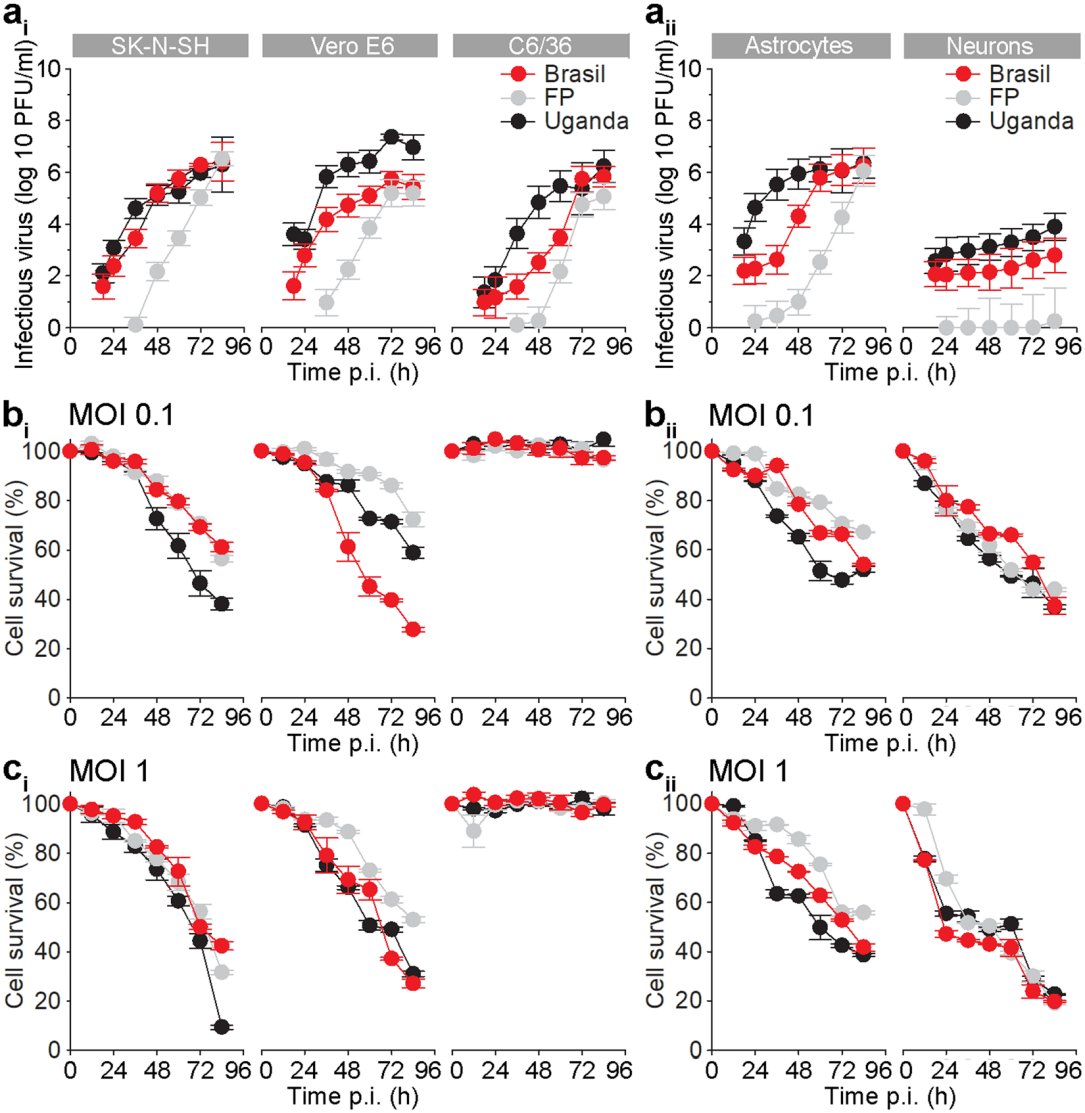
Zika Virus Production in SK-N-SH and VeroE6 Cells is Similar to C6/36 Cells which Resist Cell Death. Astrocytes Exhibit Higher Zika Virus Production than Neurons and Maintain Higher Viability. (**a**) Graphs represent ZIKV infectious virus particles in the supernatants at different hpi, as measured with the plaque assay. Trend in the production of infectious virus particles are similar in SK-N-SH, VeroE6, C6/36 cells (**a**_**i**_) and in astrocytes (**a**_**ii**_), while in neurons are lower in all three strains. The increase in infectious ZIKV-FP virus particles was the lowest in all cell types. ZIKV-FP viral particles, collected in the supernatant at 12 hpi did not produce countable plaques. (**b**) Cell survival plots for SK-N-SH, Vero E6, and mosquito C6/36 cells (**b**_**i**_) and for astrocytes and neurons (**b**_**ii**_) infected with ZIKV-UG, ZIKV-BR, and ZIKV-FP at an MOI 0.1. (**c**) Cell survival plots for SK-N-SH, Vero E6, and mosquito C6/36 cells (**c**_**i**_) and for astrocytes and neurons (**c**_**ii**_) infected with ZIKV-UG, ZIKV-BR, and ZIKV-FP at an MOI 1. Cell survival data shown in panels b and c was determined with Reliablue reagent. Noticeable cell death of all cell types, except C6/36 cells, was observed. Survival of astrocytes was higher than of neurons. Data represent means ± SEM of two independent experiments performed in triplicates in panel a, one experiment performed in triplicates in panels b_i_ and c_i_ and one experiment performed in triplicates in panels b_ii_ and c_ii_ (a total of 10^4^ cells were plated per well).

First we assessed ZIKV infection kinetics in selected mammalian SK-N-SH and Vero E6 plus C6/36 mosquito cell lines. The increase in infectious virus production was measured at an MOI 0.1 after several hours post infection (12, 18, 24, 36, 48, 60, 72, and 84 hpi). Productive ZIKV replication of strains ZIKV-BR and ZIKV-UG in the form of released infectious virions was detected at 18 hpi, while the productive replication of ZIKV-FP was delayed in all cell lines (Fig. 1a_i_). The reproducible plaque counts in all three cell lines were observed at earliest at 18 hpi, however, at 84 hpi there was no significant difference in the concentration of infectious viral particles among different strains (Fig. 1a_i_). In general, viral titers of all three ZIKV strains reached approximately 10^6^ plaque forming units (pfu)/ml in the culture medium after 72 hpi in all cell lines and remained high until the end of the experiment at 84 hpi. At 18 hpi a few plaques were produced by ZIKV-FP; however, their number was too low to accurately determine the virus titer.

Then we tested how the infectious virus production affects cell viability. We determined cell viability by two different assays; Reliablue cell viability reagent, measuring metabolic activity of living cells (Fig. 1b_i_,c_i_), and with trypan blue exclusion assay (Fig. S1). As shown in Fig. 1b_i_,c_i_, the survival of mammalian cell lines, infected with ZIKV strains at an MOI 0.1 (Fig. 1b_i_) and MOI 1 (Fig.1c_i_), were severely affected. Both mammalian cell lines, SK-N-SH and Vero E6, show somewhat similar rate of decline in cell survival at the same MOI. Among ZIKV strains it appears that ZIKV-UG affected the viability of SK-N-SH cells more than the other two strains at both tested MOIs, while in Vero E6 cells ZIKV-BR affected the viability more than ZIKV-UG and ZIKV-FP at an MOI 0.1, while at an MOI 1 only ZIKV-FP was less detrimental for cell survival. The viability of mosquito C6/36 cells was unaffected by ZIKV infection (Fig. 1b_i_,c_i_). The survival rate of cell lines measured with Reliablue reagent was higher from the one measured with trypan blue exclusion assay (Fig. S1), however, all the trends remained the same. Although the same number of cells and the same MOI (0.1) was used in both viability tests, certain differences between the two methods may be accounted to errors that may occur for instance due to poor dispersion of cells and cell loss during cell dispersion with Countess Automated Cell Counter counting. On the other hand, Reliablue Cell Viability Assay, though not error-free (e.g. possible high background in control wells), appears to be more reliable.

In general, the increase in viral production was correlated with the decrease in cell survival, except for the mosquito cells, which were resilient to ZIKV infection (Fig. 1). The time point at which the virus concentration in the supernatant reached maximal levels corresponded well with the decline in viability of all cell types (except the mosquito cell line).

### Human fetal astrocytes have higher infectious virus production and tolerate ZIKV infection better than human fetal neurons

To determine cell responses of human astrocytes and neurons to infection with selected ZIKV strains, we performed measurements of infectious viral particle production and viability of host cells, as described for cell lines. At 12 hpi we have already detected the increase in viral RNA (Fig. S1), but infectious viral particles, collected in the supernatant at this time point did not produce countable plaques (Fig. 1a_ii_). Higher quantity of infectious viral particles was observed in astrocytes (over 6-logarithmic units), further implying that astrocytes are an important source of infectious virus in the brain. On the other hand, the production of infectious virus was the lowest in neurons, where only 2-logarithmic units increase was observed (Fig. 1a_ii_). Thus, human neurons appear to be the poorest ZIKV producers among the cells tested. Additionally, we detected a difference between the growth rates among the three ZIKV strains in astrocytes and neurons, respectively. However, the overall production of ZIKV strains was lower in neurons than in astrocytes and ZIKV-FP in particular increased marginally over time in neurons. Similarly, as measured in cell lines, strains ZIKV-BR and ZIKV-UG induced the formation of the first plaques at 18 hpi, ZIKV-FP production was the slowest of all strains and there was also no significant difference in the production of infectious viral particles among the three strains at 84 hpi.

To study the effect of infectious virus production on cell viability, we infected cells with two different MOI (0.1 and 1) and measured cell survival. Both, astrocytes and neurons, survived better when they were infected at an MOI 0.1 (Fig. 1b_ii_) than at an MOI 1 (Fig. 1c_ii_). The viability of neurons was affected more than that of astrocytes. Roughly half of astrocytes were still viable at 84 hpi (MOI 0.1, ZIKV-BR, ZIKV-UG), when measured with Reliablue Cell Viability Assay. Measuring the cell survival with trypan blue exclusion assay produced slightly lower yields (Fig. S1), as observed for cell lines. At both MOI (0.1 and 1) ZIKV-FP affected cell viability of astrocytes less than ZIKV-BR and ZIKV-UG, similar as in VERO E6 cells. In neurons we did not observe any difference among strains in respect of cell survival.

Taken together, astrocytes showed similar infectious virus production as cell lines, but higher than neurons. Faster ZIKV production, especially considering the fact that astrocytes are at least as numerous as neurons^38^, speaks in favour of the hypothesis that human astrocytes are the major cell source for the spread of ZIKV through the fetal CNS.

### Different cell types show different infection rates with ZIKV; ZIKV-UG has the highest infection rate in all cell types

Once virions are adhered to the cell membrane, the virus relocates along the cell surface until it is recognized by specific binding or internalization receptors^39^. After receptor-mediated endocytosis, virions travel through the cytoplasm into which they are released to begin their replication cycle^40^. We addressed the question whether different cell types differ in the infection rate and if the infection rates are strain-specific.

**Figure 2:**
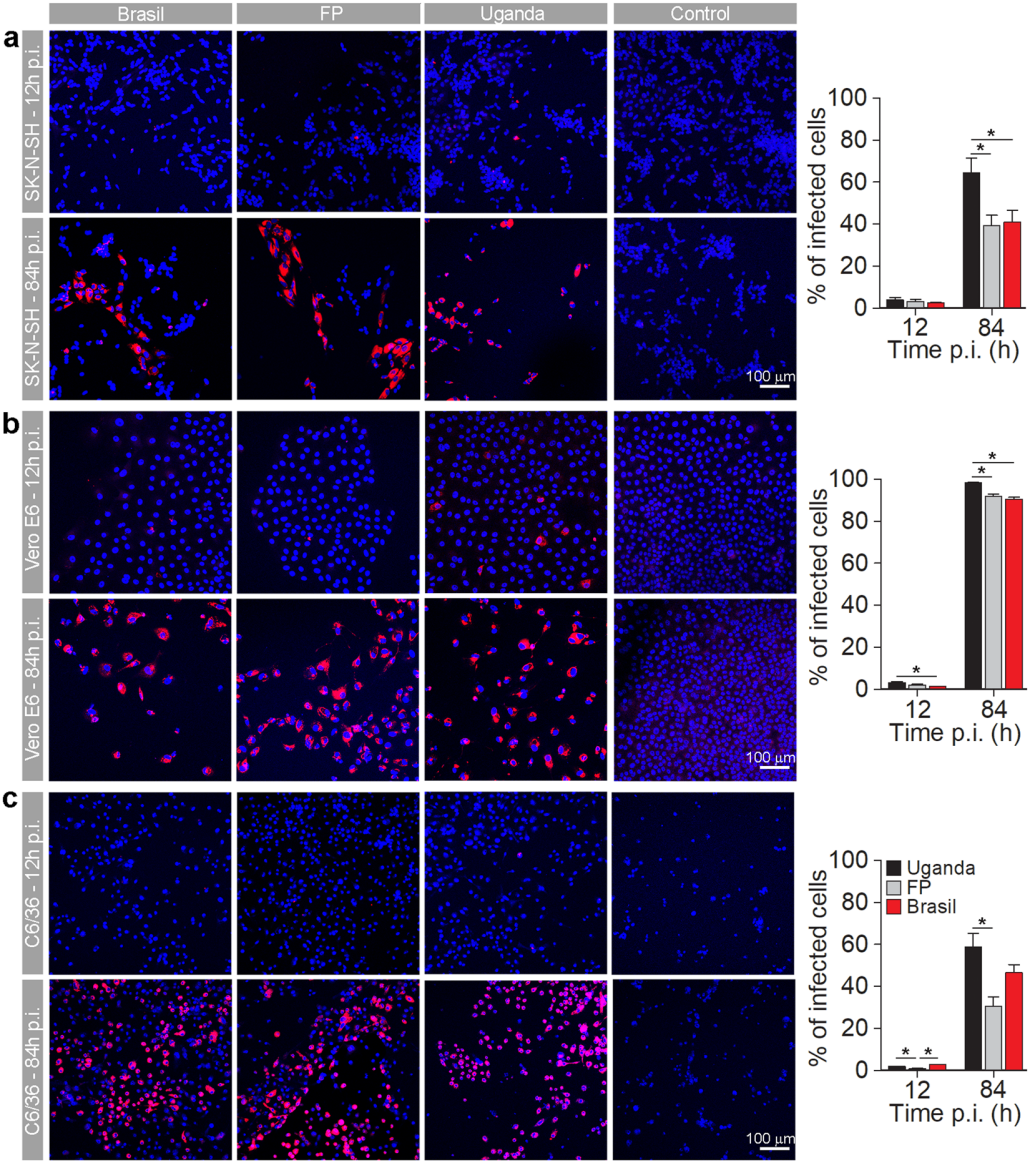
Mammalian Vero E6 cells are more efficiently infected with ZIKV than mammalian SK-N-SH and mosquito C6/36 cell lines. SK-N-SH (**a**), Vero E6 (**b**) and (**c**) C6/36 cells were infected with ZIKV-UG, ZIKV-FP and ZIKV-BR strains at an MOI 0.1 and immunolabeled with flavivirus group antigen antibodies (red) at 12 hpi and 84 hpi. Cells were also counterstained with DAPI (blue). Mock infected cells were used as negative controls (Control), labeled with primary and secondary antibodies and do not show any ZIKV labeling. The percentages of ZIKV positive SK-N-SH, Vero E6 and C6/36 cells at 12 hpi and 84 hpi are depicted in graphs (means ± SEM; One Way ANOVA, *p < 0.05). Data were collected from one experiment done in duplicates. The results are based on a total of 10^4^ cells plated per group, counted in sixteen independent fields of view (The number of cells counted per strain: 2352–4976 SK-N-SH, 1381–2947 Vero E6 and 2918–5624 C6/36). The percentage of infected cells was determined by calculating the number of immunolabeled cells versus the percentage of all DAPI stained nuclei representing single cells.

Cells were infected at an MOI 0.1 and immunolabeled against flavivirus group antigen antibodies. The infection rates in cell lines SK-N-SH, Vero E6 and C6/36 were low at 12 hpi in all three ZIKV strains tested (Fig. 2). While in SK-N-SH cells the percentages of infected cells with different ZIKV strains were similar, ZIKV-UG had significantly higher infection rate than ZIKV-BR in Vero E6 cells, and ZIKV-BR and ZIKV-UG infected significantly higher percentage of C6/36 cells than ZIKV-FP (ANOVA, p < 0.05). On the other hand, significantly higher infection rates were detected at 84 hpi, when compared to infection after 12 hpi (Mann-Whitney test; p < 0.001). ZIKV-UG induced the highest infection rates at 84 hpi in all cell lines; the highest percentage was measured in Vero E6 cells, where almost all cells were infected (Fig. 2).

**Figure 3:**
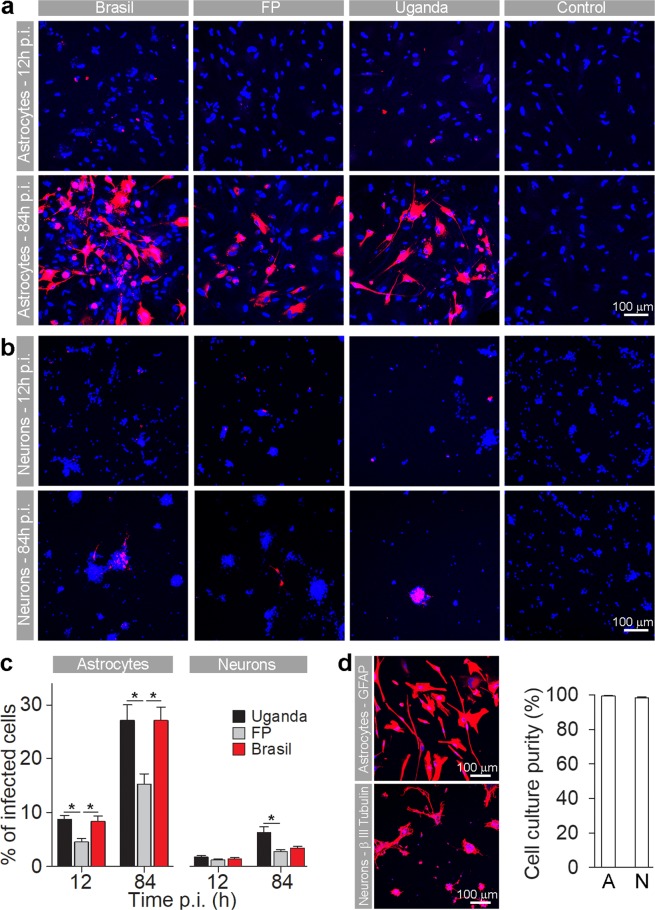
Human fetal astrocytes are more efficiently infected with ZIKV than neurons. Human fetal astrocytes (**a**) and human fetal neurons (**b**) were infected with ZIKV-UG, ZIKV-FP and ZIKV-BR strains at an MOI 0.1 and immunolabeled with flavivirus group antigen antibodies (red) at 12 hpi and 84 hpi. Cells were counterstained with DAPI (blue). Mock infected cells were used as negative controls (Control), labeled with primary and secondary antibodies and do not show any ZIKV labeling. (**c**) The percentages of ZIKV positive astrocytes and neurons at 12 hpi and 84 hpi are depicted in graphs (means ± SEM; One Way ANOVA, *p < 0.05) and they were determined by calculating the number of immunolabeled cells versus the percentage of all DAPI stained nuclei representing single cells. Data were collected from one experiment done in duplicates. Results are based on a total of 10^4^ cells plated per group, counted in sixteen independent fields of view (The number of cells counted per strain: 858–1805 astrocytes and 4882–5765 neurons). (**d**) The purity of cells cultures was tested by immunolabeling astrocytes and neurons with specific markers (GFAP and βIII Tubulin, recpectively). The percentage of GFAP positive astrocytes and βIII tubulin positive neurons were determined by calculating the number of immunolabeled cells and presented as the percentage (means ± SEM) of all cells (DAPI stained nuclei representing single cells). Cells were counted in twelve separate fields of view for each cell type (The number of cells counted per strain: 414 astrocytes and 4287 neurons), data were collected from one experiment performed in duplicates (10^4^ cells plated per sample).

In human fetal astrocytes the infection rates at 12 hpi were significantly higher than in cell lines studied (ANOVA, p < 0.05; Figs 2 and 3). At this time point, ZIKV-BR and ZIKV-UG infected significantly more astrocytes than ZIKV-FP (ANOVA, p < 0.05). At 84 hpi the percentages of infected cells were significantly higher, compared to those at 12 hpi (Mann-Whitney test; p < 0.001), however, they were lower than in cell lines at 84 hpi (ANOVA, p < 0.05; Fig. 2). Similarly, as at 12 hpi, also at 84 hpi ZIKV-BR and ZIKV-UG strains infected significantly more astrocytes than ZIKV-FP (Fig. 3). The lowest infection rates after 12 hpi were measured in human fetal neurons and they remained low after 84 hpi (Fig. 3). While there were no differences among infection rates of ZIKV strains at 12 hpi, ZIKV-UG infected significantly more neurons at 84 hpi than ZIKV-FP and ZIKV-BR (Fig. 3). To ascertain the purity of astrocyte and neuronal cultures we immunolabeled them with antibodies against astrocyte marker GFAP and neuronal marker βIII Tubulin. The results revealed that 99.3 ± 0.3% of astrocytes and 98.3 ± 0.5% of neurons were positive for respective markers (means ± SEM).

In summary, these data show that ZIKV-UG strain infected significantly more cells in the majority of cell types studied. Among five cell types tested, astrocytes showed the highest initial infection rate, while Vero E6 were the most susceptible to ZIKV infection, judging from the results at 84 hpi. Among human primary cells isolated from neural tissue, astrocytes were considerably more permissive for ZIKV infection than neurons. The difference was obvious already at 12 hpi, and increased further at 84 hpi.

### Increase in ZIKV-Laden vesicle mobility was observed in ZIKV-BR and ZIKV-UG strains

**Figure 4:**
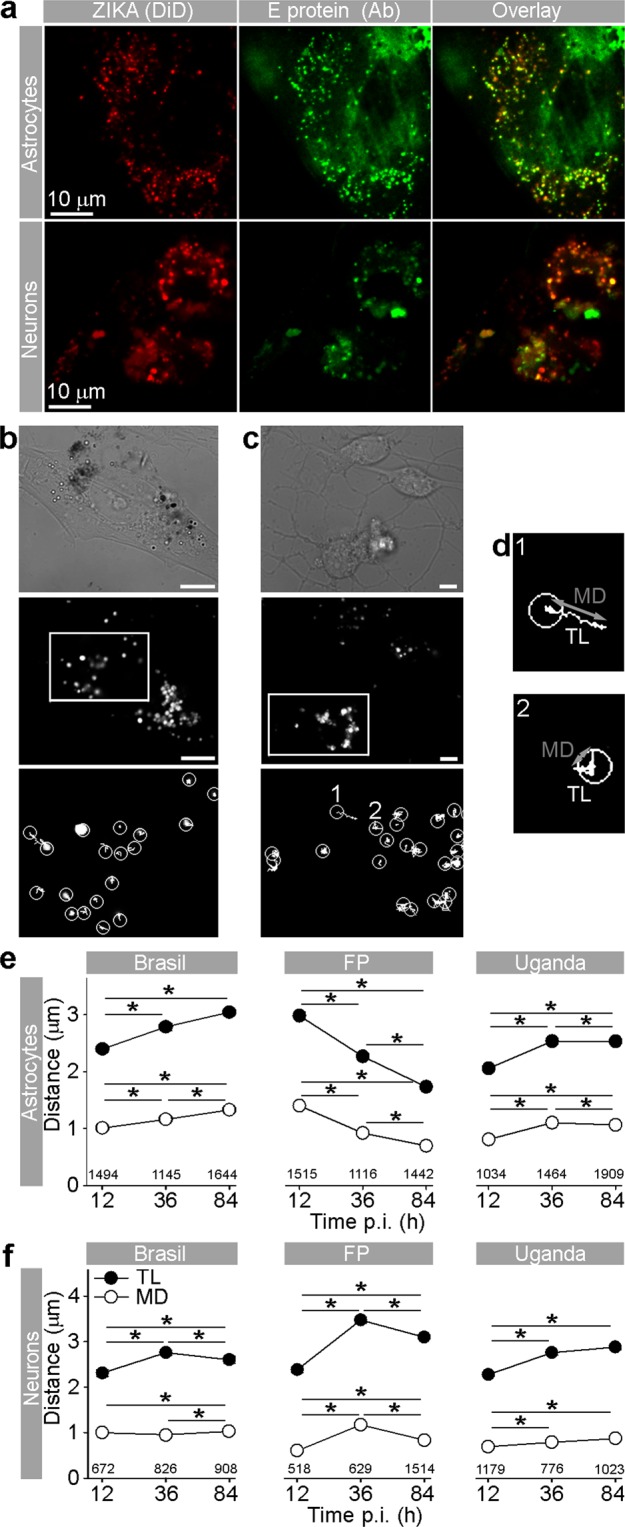
Travel Distance of Endocytotic ZIKV-Laden Vesicles in Astrocytes and Neurons Increases during Infection, with the Exception of ZIKV-FP in Astrocytes. (**a**) Fluorescent micrographs showing internalized fluorescently labeled ZIKV-BR into astrocytes and neurons (ZIKA (DiD)) and immunolabeled with serum from a patient infected with ZIKV (infected in Brazil in 2016). Overlay panels show remarkable co-localization between vesicular structures with fluorescently labeled ZIKV and anti-ZIKV antibodies from the patient’s serum. Images were recorded at 36 hpi. (**b**) An image of a ZIKV-BR-infected astrocyte at 84 hpi represented in three panels: the DIC image (upper panel), the fluorescent micrograph depicting the spotted signal of DiD-labeled ZIKV-BR vesicles (middle panel), the enlarged inset of the middle panel (rectangular area) superimposed by vesicle trajectories (lower panel). Scale bars, 10 µm. (**c**) An image of ZIKV-BR-infected neurons at 84 hpi; the DIC image of a neuronal culture (upper panel). The fluorescent micrograph depicting puncta of DiD-labeled ZIKV-BR vesicles in neurons (middle panel). The enlarged inset of the middle panel (rectangular area) superimposed by vesicle trajectories (lower panel). Vesicle trajectories labeled as 1 and 2 are expanded in (**d**). Scale bars, 5 µm. (**d**) Vesicle trajectories of the two typical trajectories noted in all cells types. The nearly straight trajectory of vesicle 1 depicts the highly directional movement of a vesicle. Travel length (TL) is similar in distance as the maximal displacement (MD) of a vesicle. The contorted trajectory of vesicle 2 depicts non-directional movement of a vesicle. TL is longer than the MD of a vesicle. Circles depict the starting positions of vesicle trajectories. TL, travel length depicted as a white trajectory; MD, maximal displacement depicted as a gray line connecting the two most distant points in a vesicle trajectory. (**e**) Graphs represent the TL and MD of vesicles in astrocytes infected with strains ZIKV-BR, ZIKV-FP, and ZIKV-UG. TL and MD of endocytotic vesicles laden with ZIKV-BR and ZIKV-UG significantly increased with longer hpi, whereas a significant decrease was observed in endocytotic vesicles laden with ZIKV-FP. The number of vesicles analyzed is denoted on the graphs. (**f**) TL and MD of neuronal endocytotic vesicles laden with ZIKV strains increased at 84 versus 12 hpi. The number of vesicles analyzed is denoted on the graphs. 17–45 cells per time period and per strain were analyzed. Data were collected from one experiment performed in duplicates and are represented as means ± SEM (One Way ANOVA, *p < 0.05).

**Figure 5:**
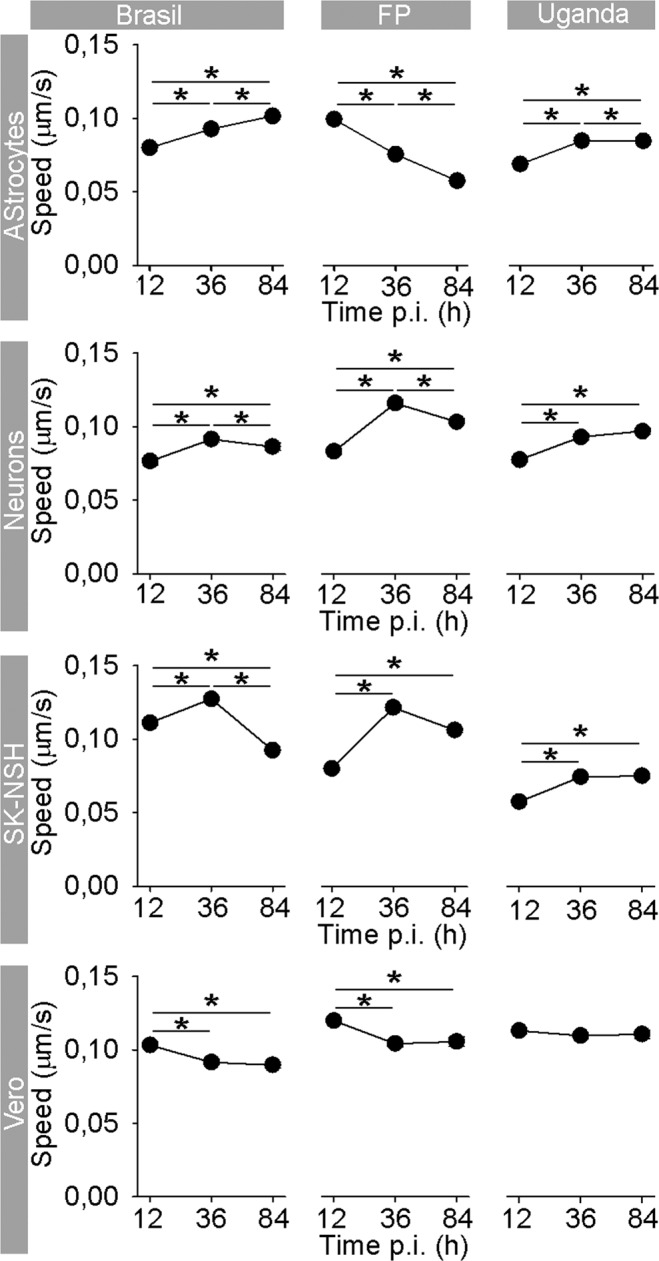
Speed of Endocytotic ZIKV-FP-Laden Vesicles Decreases the Most in Astrocytes. In astrocytes, the average speed of ZIKV-BR- and ZIKV-UG-laden vesicles significantly increased with longer hpi, whereas the speed of vesicles laden with ZIKV-FP significantly decreased. In neurons, the average vesicle speed exhibited the most prominent increase at 36 hpi, regardless of the virus strain, and then declined the most in ZIKV-FP-laden vesicles. In neuroblastoma SK-N-SH cells, the average speed of vesicles increased until 36 hpi, whereas in Vero E6 cells, the speed of virus-laden vesicles either decreased or remained unchanged. The number of analyzed cells is the same as in Figs 4, S2 and S3. Data were collected from one experiment, performed in duplicates and are represented as means ± SEM (One Way ANOVA, *p < 0.05). The average speed was calculated for the same vesicles as shown in Fig. 4.

**Figure 6:**
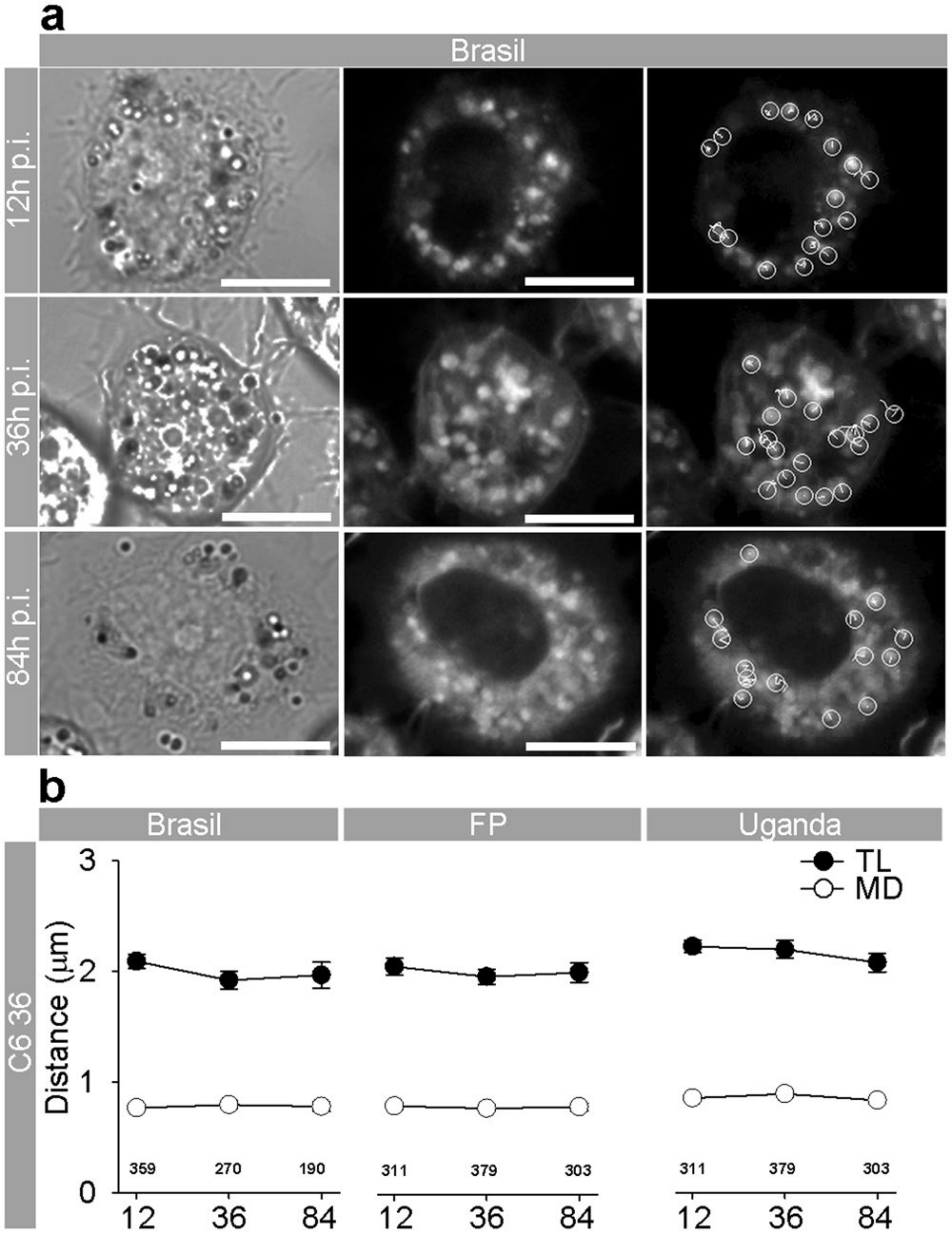
ZIKV Infection of Mosquito C6/36 Cells Does Not Affect the Mobility of DiD-ZIKV-laden vesicles. (**a**) Mosquito C6/36 cells infected with DiD-labeled ZIKV-BR at 12, 36, and 84 hpi. DIC images (left panels), fluorescent micrographs depicting punctate signals of DiD-labeled ZIKV-BR-laden vesicles (middle panels), fluorescent micrographs depicting DiD-labeled ZIKV-BR-laden vesicles with superimposed vesicle trajectories (right panels). Scale bars, 10 µm. Circles denote the starting positions of the vesicle trajectories (white lines). (**b**) Graphs represent the TL and MD of vesicles in C6/36 cells infected with strains ZIKV-BR, ZIKV-FP, and ZIKV-UG. The TL and MD of vesicles remained similar at all hpi; ZIKV-BR, p = 0.081 (TL), p = 0.532 (MD); ZIKV-FP, p = 0.767 (TL), p = 0.838 (MD); ZIKV-UG, p = 0.063 (TL), p = 0.465 (MD). The number of vesicles analyzed is denoted on the graphs. 5–9 cells per time period and per strain were analyzed. Data were collected from one experiment done in duplicates and are represented as means ± SEM (One Way ANOVA, *p < 0.05).

Upon internalization into the host cell, flaviviruses, including ZIKV, reside at first in endosomes^11,41–43^. As for their internalization, and for their delivery to replication sites, assembly, and egress, viruses use the host cytoskeleton, eventually also causing rearrangements of the cytoskeleton filaments^44^. The latter, for example, have been observed in flavivirus-infected medulloblastoma, glioblastoma, and primary astrocytes^36,45^. While still unclear, the interaction between the virus and the cytoskeleton may affect virus traffic from the plasma membrane. To test whether ZIKV strains can affect the mobility of endocytotic vesicles by which they are transported, we measured the movement of ZIKV-laden vesicles and determined the travel length (TL), net maximal displacement (MD) of vesicles and their speed. We infected cells with DiD-labeled ZIKV strains (DiD-ZIKV) and checked if the mobility of endosomes was affected after 12, 36, and 84 hpi. Inside the cells, DiD-ZIKV aggregated into vesicular structures in astrocytes and neurons (Fig. 4a). The vast majority of these intracellular fluorescent vesicular structures contained DiD-ZIKV particles and not unspecific DiD dye, that was confirmed by co-labeling with the ZIKV-antibody-positive patient’s serum (Fig. 4a). The trajectories of vesicle movements in astrocytes and neurons are represented in Fig. 4b–d. Interestingly, in astrocytes we observed differences in mobility parameters depending on the ZIKV strain (Fig. 4d). While the speed of endocytotic ZIKV-laden vesicles after 12 hpi was similar or slightly higher in comparison to the speed of endocytotic vesicles in uninfected, unstimulated, astrocytes^28,30,31,46–48^, later during the infection changes in the vesicle speed were noticed. Endocytotic vesicles loaded with ZIKV-BR or ZIKV-UG became more mobile as a function of time; TL, MD, and speed were significantly increased (Figs 4d and 5) after prolonged infection. In contrast, vesicles carrying ZIKV-FP showed progressively more confined and slower movements during the infection period; their mobility almost halved by the end of the experiment (Figs 4d and 5). In neurons, vesicles laden with ZIKV strains showed increased mobility at 84 hpi versus 12 hpi (Figs 4e and 5). ZIKV-FP- and ZIKV-UG-laden vesicles showed decreased mobility as a function of hpi only in neuronal processes (Fig. S3). In contrast, in mosquito C6/36 cells, in which ZIKV infection did not compromise survival, the trafficking dynamics of ZIKV-laden vesicles remained unaltered throughout the experiment (Fig. 6). In these cells, the intracellular signal of internalized ZIKV particles differed compared with other cell types; it was predominantly diffused with scattered assemblies of vesicular structures (Fig. 6).

In short, similarly as different ZIKV strains showed different replication dynamics in the course of the experiments (Fig. 1), we observed that different ZIKV strains also differentially affected trafficking of ZIKV-laden vesicles, an effect that was especially pronounced in astrocytes.

In addition, we analyzed vesicle trafficking of ZIKV-laden endocytotic vesicles in control cells that were used for viral amplification. SK-N-SH derived from neuroblasts in embryo or fetus^49^, Vero E6 cells and especially mosquito C6/36 cells display striking differences in response to ZIKV infection in comparison with astrocytes and neurons. In SK-N-SH and Vero E6 cells decreased mobility of endocytotic ZIKV-laden vesicles toward the end of the experiment was observed (Figs S1 and S2), while in mosquito C6/36 cells the mobility parameters were constant during the experiment.

In summary, we compared the effects of infection of the Asian-lineage ZIKV strains ZIKV-BR and ZIKV-FP; and the African-lineage strain ZIKV-UG on selected cell types. The results revealed resistance of astrocytes to ZIKV-induced cell death, considering their role in massive production of ZIKV versus neurons. Neurons proved to be the poorest producers of all ZIKV strains. In the majority of cell types, the infection rate of ZIKV-UG strain was the highest. All ZIKV strains triggered differences in trafficking of ZIKV-laden endocytotic vesicles in practically all cell types studied, except in mosquito cells, where ZIKV infection failed to induce cell death.

## Discussion

Motivated by the latest ZIKV outbreak, interest in the effects of ZIKV infection on neuronal cells, especially in the prenatal stage, has increased considerably. Several studies have addressed the issue of which cells are predominantly affected in the fetal brain. It has been shown that ZIKV infects human NPCs, NSCs, RGCs, microglial cells, oligodendrocyte precursor cells, neurons, and astrocytes^10,11,13,19,50^. However, it is unclear how infected fetal neuronal cells respond to infection with different ZIKV strains. The focus of our study was, therefore, to compare the responses of primary human fetal astrocytes and neurons to infection with low passages of the Asian-lineage strains ZIKV-BR and ZIKV-FP, and of the African-lineage strain ZIKV-UG. We also compared the responses to ZIKV infection from human astrocytes and neurons with the responses from several cell lines.

The first conclusion of this study is that astrocytes are initially more, but in the long term less susceptible to ZIKV infection than the selected mammalian and mosquito cell lines. However, astrocytes are more susceptible to ZIKV infection than neurons, as determined by the percentage of infected cells after infection with the ZIKV. On the other hand, astrocytes production of the virus was comparable to the amount produced in the selected cell lines. Considering that the production of infectious virus particles was much higher in astrocytes and their viability at least as high as in neurons, this indicates that astrocytes can tolerate higher amount of the virus load compared to neurons. The higher susceptibility of human astrocytes versus neurons is in line with the observation that ZIKV strains Cambodia (2010), Brazil (2015), and Puerto Rico (2015) infected more RGCs and astrocytes in cortical tissue slices than post-mitotic neurons in a period from early gestation to around mid-gestation^19^. Our findings further support this observation by revealing that the infection rate of ZIKV is lower in human neurons than in astrocytes. Our observation of low infection rate in human fetal astrocytes is also in line with data obtained in mice astrocytes and human adult astrocytes^51,52^. Higher infection rates in astrocytes versus neurons, regardless of the strain, reflect the differences detected in the extent and/or the types of ZIKV entry receptors described in both cell types^20^. Namely, astrocytes at mid-gestation abundantly express Axl, a type of TAM entry receptor, while NPCs and neurons poorly express Axl^20^. Higher ZIKV uptake into neurons after prolonged hpi in our experiments may indicate that infection further promotes viral entry, which *in vivo* is most likely promoted by astrocytic hyper-production of ZIKV. In line with this conclusion, we observed differences not only in the infection rate between astrocytes and neurons, but also differences in the productive virus release rate for the two cell types. It appears that in neurons, virus production and release were suppressed with prolonged infection. The fact that astrocytes apparently did not suppress virus production and proved to be much more resilient to the higher among of infectious virus particles supports the view that astrocytes are the major producer of ZIKV progeny in the brain. It is to be expected that when astrocytes become infected, massive infection of other CNS cell types will follow. From this perspective, it is of outmost importance to prevent the infection of astrocytes. This was shown to be successful with azithromycin, which reduced the infection, rescued cell viability, and decreased viral production in U87 cell lines and human pluripotent stem cell-derived astrocytes^19^.

While astrocytes are massively infected by the ZIKV strains and they vigorously produce ZIKV, they appear to be more resistant to ZIKV-induced cell death. Cell survival data of astrocytes are similar to the observation from another study where cell survival of human fetal astrocytes decreased to similar levels after 4 days post infection, however, it is noteworthy that a large proportion of astrocytes remained viable^53^. One has to be careful when comparing different viability data of human astrocytes, since there is some variability among different sources, different donors and different strains^53–55^. Astrocytes appear to be important reservoirs of ZIKV and thus are compelling targets for next-generation drug therapies. Certain pool of human fetal astrocytes can even establish persistent infection with ZIKV, further supporting the theory that these cells can serve as a reservoir for ZIKV persistence in the fetal brain^53^.

When looking at the level of different strains, it is obvious that the infection rate of ZIKV-UG was the highest in practically all cell types tested, except in human fetal astrocytes where infection rate of ZIKV-BR was similar. This observation is slightly different from the observation in human adult astrocytes, where at an MOI 0.1 African ZIKV strain infected more cells than Brasil ZIKV strain^52^. Lower infection rates at longer times post infection of ZIKV strains in astrocytes versus SK-N-SH, Vero E6 and C6/36 cells can reflect several possibilities; for example the difference in the amount of AXL surface receptor that was shown to be lower in astrocytes than in human lung carcinoma cell line^53^, or the ability of astrocytes to induce rapid upregulation of type I IFNs and thereby restricting viral replication and spread^51^. The function of Axl receptor is still debated, as whether it functions as ZIKV entry receptor or if it promotes ZIKV infection in human astrocytes by antagonizing type I IFN signaling^56^. In addition to that, virus strain itself may affect cell infection rate, as we observed in the case of ZIKV-FP, where cell infection rate was the lowest among all strains in the majority of cell types tested. Moreover, ZIKV-BR and ZIKV-UG initiated production and release of infectious virus particles more rapidly than ZIKV-FP. This finding is similar to the results of another study, in which ZIKV-FP was reported to infect a lower number of astrocytes than an ArB41644 ZIKV strain of African lineage (Bangui, Central African Republic, 1989), had lower production, and induced milder cell death^13^. Moreover, recent study showed significant differences in the growth properties of ZIKV fetal brain isolate FB-GWUH-2016 and ZIKV-FP in different cell types^57^.

In addition to the differences between strains regarding their production rate and infection, we have identified a novel property in their effect on endocytotic trafficking that differs between ZIKV strains. Whereas ZIKV-BR and ZIKV-UG strains on average enhanced endocytotic vesicle mobility in astrocytes and neurons, ZIKV-FP-laden vesicles decreased their mobility, especially in astrocytes. It remains to be highlighted in detail how specific ZIKV strains, and other flaviviruses, i.e. TBEV^36^, manipulate the trafficking machinery of endocytotic vesicles. In general, endocytotic transport can be altered by different expression of cytoskeleton proteins, Rab and Rho GTPases that regulate intracellular trafficking, and actin and microtubule assembly^36,44–46,58^. Viruses hijack a number of proteins that enable intracellular vesicle trafficking, such as actin, tubulin, dynamin, and Rho GTPases, and by their manipulation, they may alter the mobility of vesicles^44,59,60^. In addition to subverting host cell proteins, viruses also alter lipid signaling mechanisms that are responsible for entry, replication, egress and trafficking of virions in the host cell^39,61–66^. ZIKV strains may manipulate endocytotic trafficking differently through rearrangement of the cytoskeleton (as e.g., after TBEV infection) or at the transcriptional level with the upregulation of cytoskeleton-linked proteins (actin, tubulin, dynamin, and Rho GTPases) as was shown for West Nile virus^36,45,59^. Moreover, a recent study showed that genes involved in the regulation of intracellular vesicle-mediated transport are differentially expressed in human peripheral neurons infected with ZIKV strain from the 2015 Puerto Rico Zika outbreak^61^. It is hypothesized that the altered abundance of cytoskeletal proteins may participate in viral particle assembly, cargo, and egress, leading to the replication and release of mature virions^59^.

In conclusion, this study highlights the potential of astrocytes to be the main ZIKV source in the CNS, as they massively produce and release ZIKV, and are resilient to a high virus production. Neurons, on the other hand, appear to suppress the production of virus and also have lower infection rates. Variabilities among different ZIKV strains are visible at several steps of the viral cycle, such as different production and infection rates, infection and trafficking of endocytotic ZIKV-laden vesicles. Future studies are needed to highlight the strain-specific relationships between the cytoskeleton and altered vesicle trafficking.

## Methods

### Cell cultures

Human fetal cortical astrocytes (20 gw) were obtained from Innoprot (Derio, Spain). They were cryopreserved at passage one and checked for cell culture purity by GFAP immunofluorescent staining by the provider prior to shipment. Upon delivery they were grown for one passage (4 days) in cell-culture flasks and plated onto chambered coverslips (µ-Slide 2 Well; Ibidi, Martinsried) for use in the experiments within the following three days. They were maintained in complete astrocyte medium (Innoprot) at 37 °C in 5% CO_2_.

Human fetal neurons (20 gw), cryopreserved immediately after tissue dissociation, at passage 0 (P0), were acquired from Innoprot (Spain). Neurons were thawed and plated directly onto poly-D-lysine (Sigma)-coated chambered coverslips (µ-Slide 2 Well; Ibidi, Germany) for use in the experiments within the following three days. They were maintained in complete neuronal medium (Innoprot) at 37 °C in 5% CO_2_/95% air.

Human neuroblastoma, SK-N-SH (ATTC HTB-11), and African green monkey kidney cells, Vero E6 cells (ATTC CRL-1586), were grown in DMEM GlutaMAX (DMEM with GlutaMAX Supplement; Thermo Fisher Scientific) supplemented with 10% fetal bovine serum (FBS; Thermo Fisher Scientific) at 37 °C in 5% CO_2_.

Mosquito *Aedes albopictus*, C6/36 cells^67^ (kindly provided by Dr. David H. Walker, University of Texas Medical Branch, Department of Pathology, Galveston, Texas, USA), were grown in Leibovitz’s L-15 medium with GlutaMAX supplemented with 10% FBS at 28 °C.

### ZIKV Strains and cellular infection

ZIKV strains #976 (historical strain, isolated in 1947 in Uganda; passaged 3 × in baby mouse brain, then lyophilised in 1961; ZIKV-UG), H/PF/2013 (isolated from patient’s serum from the French Polynesia outbreak in 2013; ZIKV-FP; the strain was obtained from the EVAg collection no.001 V-EVA1545 (UMR 190-Unite Des Virus Emergentes, Marseille, France, five passages on Vero E6 cells), and BR/800/16 (isolated in Slovenia from patient returning from Brazil in May 2016; ZIKV-BR)^4,68,69^. Before the experiment, all strains were propagated in Vero E6 in DMEM GlutaMAX with 4% FBS to prepare virus stocks: ZIKV-UG, three passages; ZIKV-BR, three passages; ZIKV-FP, two passages. After harvesting the virus, the cell suspension was centrifuged at 4 °C/1,000 × *g* for 5 min and the supernatant was collected into aliquots.

For the experiments, all cells were plated onto two-chambered coverslips (µ-Slide 2 Well; Ibidi, Germany, Martinsried) 48 h before infection with ZIKV strains (unlabeled and DiD-labeled) at an MOI of 0.1. Before inoculation, cells were washed twice with sterile PBS, and ZIKV was added to the cells in a low medium volume (600 µL). ZIKV was allowed to adsorb to an 80% confluent cell monolayer for 1 h at 37 °C or 28 °C (C6/36). Following adsorption, the monolayers were washed with PBS twice and grown in fresh growth medium. Control samples were incubated with the corresponding culture growth media (mock condition). Supernatants for RT-qPCR and plaque assay were collected at 12, 18, 24, 36, 48, 60, 72, and 84 h post infection (hpi), and cell viability was assessed either with a trypan blue exclusion test on Countess Automated Cell Counter (Invitrogen) or a Reliablue Cell Viability Reagent (ATCC 30-1014) that measures cell metabolic activity. In brief, cells were plated at the concentration of 10^4^ cells/100 μl/well in a 96 well plate, 24 h before inoculation. At each inoculation time point (12, 18, 24, 36, 48, 60, 72, and 84 h) the medium was removed, cells were washed with PBS twice, and 100 µL of the virus was inoculated. At 84 h post infection 10 µL of Reliablue reagent was added with a multichannel pipet. After 1 h incubation at 37 °C (or 28 °C for C6/36) a fluorescent signal (excited at 540 nm and emission gathered at 600 nm) was read on the fluorescence plate reader (Cytation 3 Cell Imaging Reader, BioTek). Each plate included several control points: wells with only medium (background), wells with non-infected cells (negative control) and cells treated with 1% Triton (positive control). To account for the background, the average RFU values of the medium-only wells were subtracted from the results. To calculate the percentage of viability, each infection time point was plotted against negative control. All experiments were performed in triplicates in two individual experiments with MOI 0.1 or MOI 1.

To monitor internalization and endocytotic vesicle mobility, DiD-labeled ZIKV strains were applied to cells at 4 °C for 10 min to synchronize virus infection and then incubated at 37 °C^70^. ZIKV was allowed to adsorb for 1 h at 37 °C or 28 °C (C6/36), then the cells were washed with PBS twice and grown in fresh growth medium.

### RT-qPCR

Supernatants for RT-qPCR were collected at 12, 18, 24, 36, 48, 60, 72, and 84 hpi. Total nucleic acid was extracted from the cell-culture supernatants using an EZ1 Virus Mini Kit v2.0 (QIAGEN, Hilden, Germany) following the manufacturer’s instructions. Briefly, 400 µL of supernatant was used for the extraction and was eluted into 60 µL of AVE buffer. For quantification of ZIKV RNA we used two different quantitative RT-PCRs: a RealStar Zika Virus RT-PCR Kit 1.0 (Altona Diagnostics, Hamburg, Germany) and LightMix Modular Zika Virus Assay (Roche, Basel, Switzerland) on an ABI7500 Fast platform (Thermo Fischer Scientific). All molecular tests were performed in duplicates, including 10-fold standard curve of WHO Zika virus standard.

### Plaque assay

Supernatants were collected at 12, 18, 24, 36, 48, 60, 72, and 84 hpi and titrated by plaque assay according to the previously described protocol^71^. Briefly, Vero E6 cells were plated at the concentration of 4 × 10^4^ cells/500 μl/well in a 24, well plate, 24 h before inoculation. The medium was removed, cells were washed with PBS twice, and incubated with serial dilutions of collected supernatants, in medium without FBS. After 90-min incubation under slight agitation, medium was removed, cells were washed with PBS twice, and cultured with 1.5% carboxy-methil-cellulose (CMC), supplemented with 2% FCS. After 5 days, cells were fixed overnight with 4% formaldehyde and stained with 1% violet crystal for 1 h. Plaques were counted and virus yield was calculated and expressed as PFU/ml. All experiments were performed in triplicates.

### ZIKV labeling

ZIKV labeling was performed as described^36^. Briefly, ZIKV strains were grown for 4 days on Vero E6 cells. The supernatant was centrifuged twice at +4 °C (10 min at 3,200 × *g* and 5 min at 20,800 × *g*) to ensure that all cells were removed from the supernatant. The supernatant was then mixed with 50 µM fluorescent lipophilic Vybrant DiD labeling solution (DiD; Molecular Probes, Invitrogen) and incubated for 2 hr at 37 °C. The unbound dye was removed by buffer exchange into Hepes 145 buffer (50 mM Hepes, 145 mM NaCl [pH 7.4] in Illustra NAP-5 columns; GE Healthcare). The infectivity of DiD labeled viruses was verified with plaque assay according to the protocol described above. The DiD labeling did not influence virus infectivity. Labeled virus was diluted to a concentration of 5.0 log_10_ PFU/mL and stored at −80 °C until the infection experiments.

### Immunocytochemistry

Cells were washed with PBS, fixed in 4% formaldehyde in PBS for 10 min at room temperature (RT), and permeabilized with Triton X-100 for 10 min at RT. Non-specific background staining was reduced by incubating the cells in blocking buffer containing 3% BSA and 10% goat serum in PBS at 37 °C for 1 hr. The infection with DID-labeled ZIKV was confirmed by staining the cells with serum from a patient infected with ZIKV (infected in Brazil in 2016) (1:128 diluted in PBS) and incubated at 37 °C for 30 min. Afterwards, the cells were rinsed in PBS and stained with secondary antibodies (1:200 Fluoline G, BioMerieux, Lyon, France) at 37 °C for 30 min.

The purity of human astrocyte and human neuronal cultures was confirmed by labeling with primary mouse monoclonal antibodies against GFAP (1:200; Sigma) and neuron-specific β-III Tubulin (1:100; Chemicon) followed by secondary antibodies Alexa Fluor 564-conjugated anti-mouse IgG (1:600, Life Technologies). Primary and secondary antibodies were diluted in 3% PBS/BSA and incubated on cells for 2 h (primary) or 45 min (secondary) at 37 °C. At the end of the immunocytochemistry staining protocols, the cells were washed in PBS and covered with Slowfade Gold antifade reagent (Molecular Probes, Invitrogen).

The percentage of ZIKV infected cells was determined by immunolabeling the infected cells with primary mouse monoclonal anti-flavivirus group antigen antibodies (1:600; clone D1-4G2-4-15; Sigma) and secondary Alexa Fluor 564-conjugated anti-mouse IgG antibodies (1:600, Life Technologies). Primary and secondary antibodies were diluted in 3% PBS/BSA and incubated on cells for 2 h (primary) or 45 min (secondary) at 37 °C. At the end of the immunocytochemistry staining, the cells were washed in PBS and covered with SlowFade Gold Antifade Mountant with DAPI (Thermo Fisher Scientific).

### Imaging

Imaging of fixed and live cells was performed with a laser confocal microscope (LSM 780, Zeiss, Germany) using air (20×/NA 0.8 M27) and oil-immersion objectives (40×/NA 1.3 and 63×/NA 1.4). A He/Ne laser was used (633 nm) for excitation of DiD dye; the emission light was filtered with a long-pass filter with the cut off below 650 nm. Fluorescein isothiocyanate (FITC) conjugate was excited by argon laser (488 nm), and the emission light was collected through a band-pass filter (505–530 nm). Alexa Fluor 564 dye was excited with the He/Ne laser (546 nm), and the emission light was filtered with a long-pass filter with a cut-off below 560 nm. DAPI was excited with the diod laser (405 nm) and the emission light was filtered with a band-pass filter (417–477 nm). In live cells, the mobility of vesicles that expressed DiD fluorescence of ZIKV was recorded. Time-series images were recorded at 2 s intervals for a total recording time of 2 min.

### Analysis

The mobility of fluorescently labeled ZIKV-containing vesicles was analyzed by ParticleTR software (Celica Biomedical, Slovenia). Vesicle mobility parameters, travel length (TL, the total length of the vesicle pathway), maximal displacement (MD, maximal net distance of the vesicle movement), and speed were calculated as described^72^. The analysis of the vesicle mobility was performed for epochs of 30 s.

### Statistical analysis

Statistical analysis was determined with the ANOVA test in Sigma Plot software; data in the figures are presented as means ± SEM.

## Supplementary information

Dataset 1

## References

[1] Musso D, Baud D, Gubler DJ (2016). Zika virus: what do we know?. Clin Microbiol Infect.

[2] Baud, D., Musso, D., Vouga, M., Alves, M. P. & Vulliemoz, N. Zika virus: A new threat to human reproduction. *Am J Reprod Immunol* (2016). 10.1111/aji.12614 27966802

[3] Cao-Lormeau VM (2014). Zika virus, French polynesia, South pacific, 2013. Emerg Infect Dis.

[4] Mlakar J (2016). Zika Virus Associated with Microcephaly. N Engl J Med.

[5] Melo AS (2016). Congenital Zika Virus Infection: Beyond Neonatal Microcephaly. JAMA Neurol.

[6] Driggers RW (2016). Zika Virus Infection with Prolonged Maternal Viremia and Fetal Brain Abnormalities. N Engl J Med.

[7] Brasil P (2016). Zika Virus Infection in Pregnant Women in Rio de Janeiro. N Engl J Med.

[8] Martines RB (2016). Notes from the Field: Evidence of Zika Virus Infection in Brain and Placental Tissues from Two Congenitally Infected Newborns and Two Fetal Losses–Brazil, 2015. MMWR Morb Mortal Wkly Rep.

[9] Martines RB (2016). Pathology of congenital Zika syndrome in Brazil: a case series. Lancet.

[10] Li C (2016). Zika Virus Disrupts Neural Progenitor Development and Leads to Microcephaly in Mice. Cell Stem Cell.

[11] Meertens L (2017). Axl Mediates ZIKA Virus Entry in Human Glial Cells and Modulates Innate Immune Responses. Cell Rep.

[12] Hamel R (2015). Biology of Zika Virus Infection in Human Skin Cells. J Virol.

[13] Simonin Y (2016). Zika Virus Strains Potentially Display Different Infectious Profiles in Human Neural Cells. EBioMedicine.

[14] Wu KY (2016). Vertical transmission of Zika virus targeting the radial glial cells affects cortex development of offspring mice. Cell Res.

[15] Zecevic N (2004). Specific characteristic of radial glia in the human fetal telencephalon. Glia.

[16] Zecevic N, Chen Y, Filipovic R (2005). Contributions of cortical subventricular zone to the development of the human cerebral cortex. J Comp Neurol.

[17] deAzevedo LC (2003). Cortical radial glial cells in human fetuses: depth-correlated transformation into astrocytes. J Neurobiol.

[18] Hansen DV, Lui JH, Parker PR, Kriegstein AR (2010). Neurogenic radial glia in the outer subventricular zone of human neocortex. Nature.

[19] Retallack H (2016). Zika virus cell tropism in the developing human brain and inhibition by azithromycin. Proc Natl Acad Sci USA.

[20] Nowakowski TJ (2016). Expression Analysis Highlights AXL as a Candidate Zika Virus Entry Receptor in Neural Stem Cells. Cell Stem Cell.

[21] Alvarez JI, Katayama T, Prat A (2013). Glial influence on the blood brain barrier. Glia.

[22] Parpura V (2012). Glial cells in (patho)physiology. J Neurochem.

[23] Haydon P (2001). GLIA: listening and talking to the synapse. Nat Rev Neurosci.

[24] Parpura, V., Baker, B., Jeras, M. & Zorec, R. Regulated exocytosis in astrocytic signal integration. *Neurochem Int* (2010). 10.1016/j.neuint.2010.02.007 PMC2892557 20156504

[25] Zonta M (2003). Neuron-to-astrocyte signaling is central to the dynamic control of brain microcirculation. Nat Neurosci.

[26] Sultan S (2015). Synaptic Integration of Adult-Born Hippocampal Neurons Is Locally Controlled by Astrocytes. Neuron.

[27] Stenovec M (2016). Expression of familial Alzheimer disease presenilin 1 gene attenuates vesicle traffic and reduces peptide secretion in cultured astrocytes devoid of pathologic tissue environment. Glia.

[28] Potokar M, Stenovec M, Kreft M, Gabrijel M, Zorec R (2011). Physiopathologic dynamics of vesicle traffic in astrocytes. Histol Histopathol.

[29] Vardjan N, Verkhratsky A, Zorec R (2015). Pathologic potential of astrocytic vesicle traffic: new targets to treat neurologic diseases?. Cell Transplant.

[30] Potokar M (2013). Astrocytic vesicle mobility in health and disease. Int J Mol Sci.

[31] Vardjan N (2012). IFN-γ-induced increase in the mobility of MHC class II compartments in astrocytes depends on intermediate filaments. J Neuroinflammation.

[32] Potokar M (2017). Impaired αGDI Function in the X-Linked Intellectual Disability: The Impact on Astroglia Vesicle Dynamics. Mol Neurobiol.

[33] Sager, G., Gabaglio, S., Sztul, E. & Belov, G. A. Role of Host Cell Secretory Machinery in Zika Virus Life Cycle. *Viruses* **10** (2018). 10.3390/v10100559 PMC6213159 30326556

[34] Cortese M (2017). Ultrastructural Characterization of Zika Virus Replication Factories. Cell Rep.

[35] Knap N (2012). Patterns of tick-borne encephalitis virus infection in rodents in Slovenia. Vector Borne Zoonotic Dis.

[36] Potokar M, Korva M, Jorgacevski J, Avsic-Zupanc T, Zorec R (2014). Tick-borne encephalitis virus infects rat astrocytes but does not affect their viability. PLoS One.

[37] Palus M (2014). Infection and injury of human astrocytes by tick-borne encephalitis virus. J Gen Virol.

[38] Verkhratsky A, Zorec R, Parpura V (2017). Stratification of astrocytes in healthy and diseased brain. Brain Pathol.

[39] Mazzon M, Mercer J (2014). Lipid interactions during virus entry and infection. Cell Microbiol.

[40] Mandl C (2005). Steps of the tick-borne encephalitis virus replication cycle that affect neuropathogenesis. Virus Res.

[41] Chu J, Ng M (2004). Infectious entry of West Nile virus occurs through a clathrin-mediated endocytic pathway. J Virol.

[42] Mosso C, Galván-Mendoza IJ, Ludert JE, del Angel RM (2008). Endocytic pathway followed by dengue virus to infect the mosquito cell line C6/36 HT. Virology.

[43] van der Schaar HM (2008). Dissecting the cell entry pathway of dengue virus by single-particle tracking in living cells. PLoS Pathog.

[44] Taylor MP, Koyuncu OO, Enquist LW (2011). Subversion of the actin cytoskeleton during viral infection. Nat Rev Microbiol.

[45] Růzek D (2009). Morphological changes in human neural cells following tick-borne encephalitis virus infection. J Gen Virol.

[46] Potokar M, Lacovich V, Chowdhury HH, Kreft M, Zorec R (2012). Rab4 and Rab5 GTPase are required for directional mobility of endocytic vesicles in astrocytes. Glia.

[47] Potokar M (2013). Regulation of AQP4 surface expression via vesicle mobility in astrocytes. Glia.

[48] Wilhelmsson U (2012). Astrocytes negatively regulate neurogenesis through the Jagged1-mediated Notch pathway. Stem Cells.

[49] Marshall GM (2014). The prenatal origins of cancer. Nat Rev Cancer.

[50] Tang Y (2015). Mertk deficiency affects macrophage directional migration via disruption of cytoskeletal organization. PLoS One.

[51] Lindqvist R (2016). Fast type I interferon response protects astrocytes from flavivirus infection and virus-induced cytopathic effects. J Neuroinflammation.

[52] Stefanik M (2018). Characterisation of Zika virus infection in primary human astrocytes. BMC Neurosci.

[53] Limonta Daniel, Jovel Juan, Kumar Anil, Airo Adriana, Hou Shangmei, Saito Leina, Branton William, Ka-Shu Wong Gane, Mason Andrew, Power Christopher, Hobman Tom (2018). Human Fetal Astrocytes Infected with Zika Virus Exhibit Delayed Apoptosis and Resistance to Interferon: Implications for Persistence. Viruses.

[54] Xu M (2016). Identification of small-molecule inhibitors of Zika virus infection and induced neural cell death via a drug repurposing screen. Nat Med.

[55] Monel B (2017). Zika virus induces massive cytoplasmic vacuolization and paraptosis-like death in infected cells. EMBO J.

[56] Chen J (2018). AXL promotes Zika virus infection in astrocytes by antagonizing type I interferon signalling. Nat Microbiol.

[57] Kuivanen S (2017). Differences in the growth properties of Zika virus foetal brain isolate and related epidemic strains *in vitro*. J Gen Virol.

[58] Horgan CP, McCaffrey MW (2011). Rab GTPases and microtubule motors. Biochem Soc Trans.

[59] Fraisier C (2013). Altered protein networks and cellular pathways in severe west nile disease in mice. PLoS One.

[60] Foo KY, Chee HY (2015). Interaction between Flavivirus and Cytoskeleton during Virus Replication. Biomed Res Int.

[61] Oh, Y. *et al*. Zika virus directly infects peripheral neurons and induces cell death. *Nat Neurosci* (2017). 10.1038/nn.4612 PMC5575960 28758997

[62] Heaton NS, Randall G (2011). Multifaceted roles for lipids in viral infection. Trends Microbiol.

[63] Diamond DL (2010). Temporal proteome and lipidome profiles reveal hepatitis C virus-associated reprogramming of hepatocellular metabolism and bioenergetics. PLoS Pathog.

[64] Martín-Acebes MA, Vázquez-Calvo Á, Saiz JC (2016). Lipids and flaviviruses, present and future perspectives for the control of dengue, Zika, and West Nile viruses. Prog Lipid Res.

[65] Martín-Acebes MA (2014). The composition of West Nile virus lipid envelope unveils a role of sphingolipid metabolism in flavivirus biogenesis. J Virol.

[66] Perera R (2012). Dengue virus infection perturbs lipid homeostasis in infected mosquito cells. PLoS Pathog.

[67] Igarashi A (1978). Isolation of a Singh’s Aedes albopictus cell clone sensitive to Dengue and Chikungunya viruses. J Gen Virol.

[68] Wang L (2016). From Mosquitos to Humans: Genetic Evolution of Zika Virus. Cell Host Microbe.

[69] Faye O (2014). Molecular evolution of Zika virus during its emergence in the 20(th) century. PLoS Negl Trop Dis.

[70] Lakadamyali M, Rust M, Babcock H, Zhuang X (2003). Visualizing infection of individual influenza viruses. Proc Natl Acad Sci USA.

[71] Coelho SVA (2017). Development of standard methods for Zika virus propagation, titration, and purification. J Virol Methods.

[72] Potokar M, Kreft M, Pangrsic T, Zorec R (2005). Vesicle mobility studied in cultured astrocytes. Biochem Biophys Res Commun.

