# Supplementary material for: ZIKV Strains Differentially Affect Survival of Human Fetal Astrocytes versus Neurons and Traffic of ZIKV-Laden Endocytotic Compartments
Source: Sci Rep. 2019 May 30;9:8069. doi: 10.1038/s41598-019-44559-8 (PMC6542792; doi:10.1038/s41598-019-44559-8)
Supplement: Supplementary file 1 — Dataset 1 [file 41598_2019_44559_MOESM1_ESM.pdf]

ZIKV Strains Differentially Affect Survival of Human Fetal Astrocytes versus Neurons and Traffic of ZIKV-Laden Endocytotic Compartments – supplementary information

Jernej Jorgačevski[1,3,4], Miša Korva[2,4], Maja Potokar[1,3,4], Marjeta Lisjak[1], Tatjana Avšič-Županc[2,]*, Robert Zorec[1,3,5,]**

[1]Laboratory of Neuroendocrinology – Molecular Cell Physiology, Institute of Pathophysiology, Faculty of Medicine, University of Ljubljana, Zaloška 4, 1000 Ljubljana, Slovenia

[2]Institute of Microbiology and Immunology, Faculty of Medicine, University of Ljubljana, Zaloška 4, 1000 Ljubljana, Slovenia

[3]Celica BIOMEDICAL, Tehnološki park 24, 1000 Ljubljana, Slovenia

[4]These authors contributed equally

[5]Lead Contact

*Correspondence: tatjana.avsic@mf.uni-lj.si

**Correspondence: robert.zorec@mf.uni-lj.si

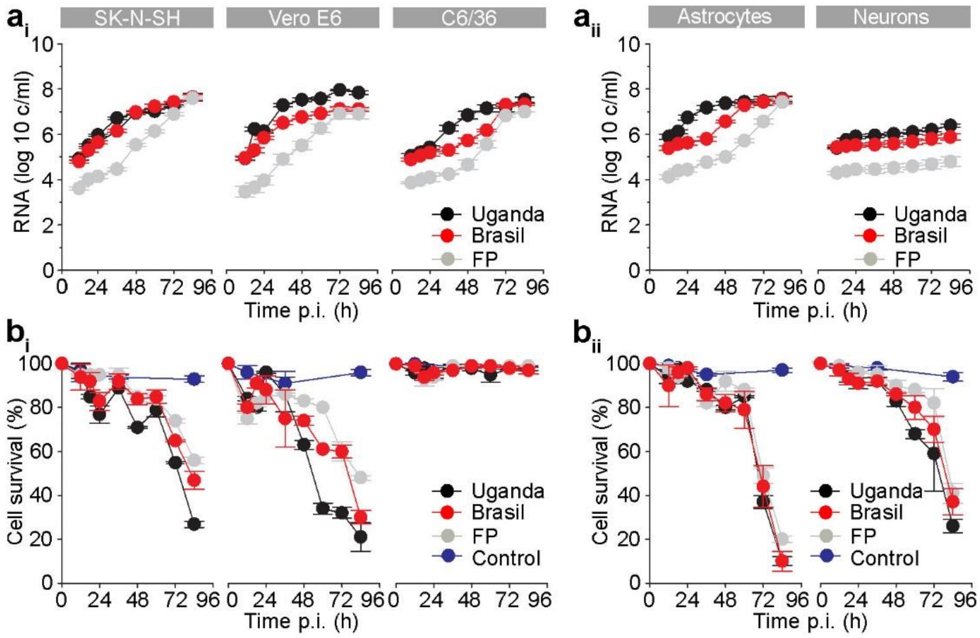

Figure S1. Zika Virus RNA Release is similar among SK-N-SH, Vero E6 and C6/36 Cells. Astrocytes Reach Higher Zika Virus RNA release than Neurons.

(**a**) Graphs represent ZIKV RNA copies in the supernatant at different hpi in SK-N-SH, Vero E6 and C6/36 cells ($\mathbf{a}_{\mathbf{i}}$) and in astrocytes and neurons ($\mathbf{a}_{\mathbf{ii}}$). The concentration of ZIKV RNA was measured using one-step quantitative real time RT-PCR.

(**b**) Cell survival plots for SK-N-SH, Vero E6 and mosquito C6/36 cells ($\mathbf{b}_{\mathbf{i}}$) and for astrocytes and neurons ($\mathbf{b}_{\mathbf{ii}}$) infected with ZIKV-UG (Uganda), ZIKV-BR (Brasil), and ZIKV-FP (FP) at an MOI 0.1. Cell survival was determined with a trypan blue exclusion test on Countess Automated Cell Counter. Cell death of all cell types, except C6/36 cells, was observed. Cell survival data measured with a trypan blue exclusion test are to a certain extent lower that data obtained by Reliablue Cell Viability Assay that measures metabolic activity of cells (Fig 1). Data represent means ± SEM of one independent experiment performed in duplicates (a total of 10,000 cells were plated per well).

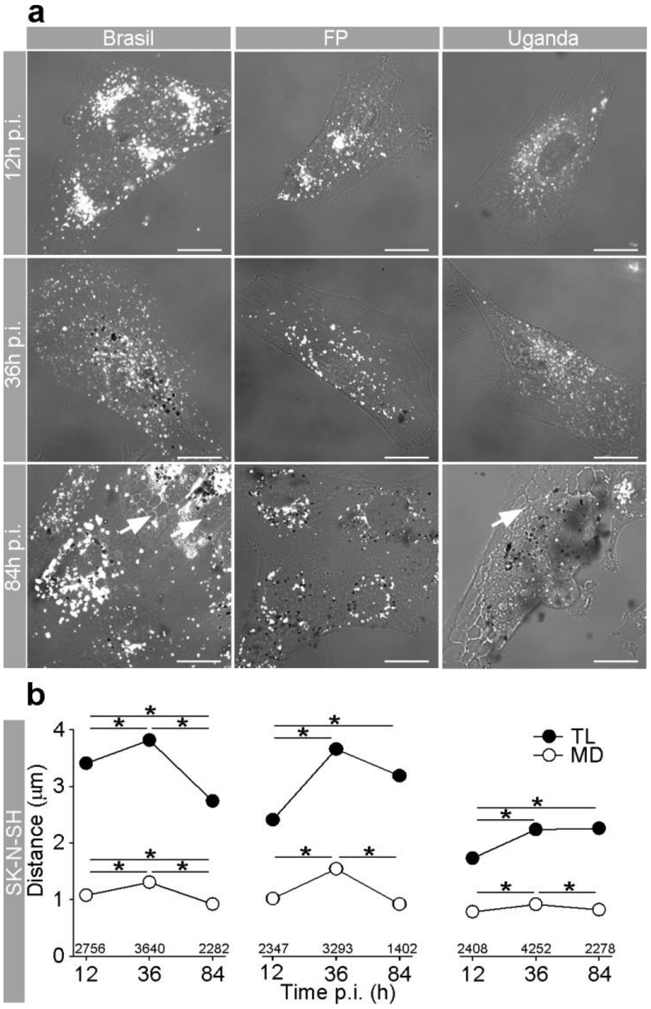

Figure S2. ZIKV Infection Transiently Increases the Travel Length and Maximal Displacement of Vesicle Trajectories in SK-N-SH Neuroblastoma Cells.

(**a**) DIC images of SK-N-SH cells infected with strains ZIKV-BR, ZIKV-FP, and ZIKV-UG. ZIKV-laden endocytotic vesicles are seen as white dots. At 84 hpi, extensive cytomorphological changes (CPE) developed, and were the most obvious in cells infected with ZIKV-BR and ZIKV-UG, as pinpointed by the arrows. Scale bars, 20 µm. (**b**) TL and MD of ZIKV-laden endocytotic vesicles increased until 36 hpi, and then declined (with the exception of the TL of ZIKV-UG-laden vesicles). The number of vesicles analyzed is indicated on the graphs. 6-9 cells per time period and per strain were analyzed. A total of 10,000 cells were plated per well; data were collected from

one experiment performed in duplicates and are represented as means ± SEM (One Way ANOVA, $*p < 0.05$).

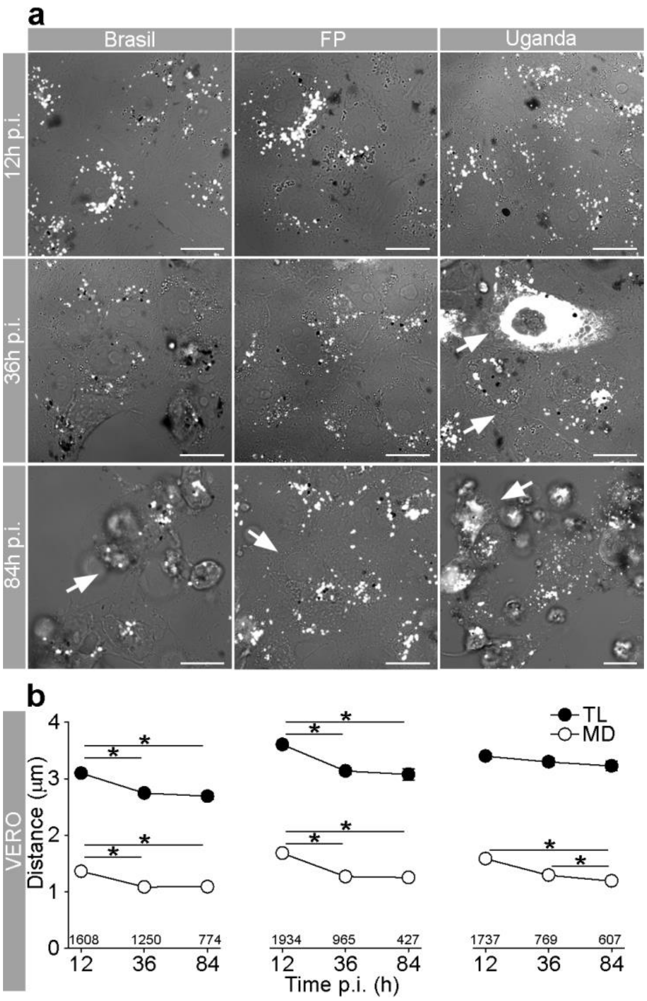

Figure S3. ZIKV Infection Attenuates Vesicle Trafficking in Vero E6 Cells.

(**a**) DIC images of Vero E6 cells infected with strains ZIKV-BR, ZIKV-FP, and ZIKV-UG. ZIKV-laden endocytotic vesicles are seen as white dots. At 84 hpi, extensive cytopathological changes developed in cells infected with either strain, and in ZIKV-UG infected cells already at 36 hpi. Cytopathological changes are pinpointed by arrows. Scale bars, 20 µm. **(b**) TL and MD of ZIKV-laden endocytotic vesicles declined with increasing hpi. The number of vesicles analyzed is indicated on the graphs. 10-12 cells per time period and per strain were analyzed. A total of 10,000

cells were plated per well; data were collected from one experiment performed in duplicates and are represented as means ± SEM (One Way ANOVA, $*p < 0.05$).

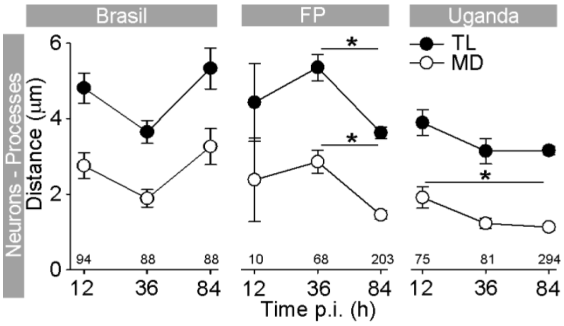

Figure S4. ZIKV-FP and ZIKV-UG Infection of Neurons Attenuate Trafficking of ZIKV-Laden Endocytic Vesicles in Neuronal Processes.

TL and MD of ZIKV-FP- and ZIKV-UG-laden endocytotic vesicles decreased with increasing hpi. The number of vesicles analyzed is indicated on the graphs. 17-45 cells per time period and per strain were analyzed. A total of 10,000 cells were plated per well; data were collected from one experiment performed in duplicates and are represented as means ± SEM (One Way ANOVA, *$p < 0.05$).
